# Segmented therapy in surgery

**DOI:** 10.1186/1746-160X-10-S1-O4

**Published:** 2014-12-12

**Authors:** Konrad Wangerin

**Affiliations:** 1 Paracelsus-Krankenhaus Ruit - Klinik für Gesichts-, Kiefer- und Wiederherstellungschirurgie, Ostfildern, Germany

## 

The combined treatment of severe dentofacial deformities normally consists of at least three steps: The pre- and post-operative orthodontic treatment and the treatment between surgical correction. The accuracy of the surgical planning and especially the surgery itself are crucial for the duration of the postoperative orthodontic treatment. Main reason for the segmentation of the jaws is the acceleration of the orthodontic treatment.

In our of 195 bimaxillary cases from three years we have observed the following indications for maxillary segmentation:

• Frontal open bite.

• Pro- or retrusion of the incisors.

• Bolton discrepancies.

• Asymmetry of the dental arch.

During LeFort I osteotomy we performed the partition in two, three or four parts. We observed the following indications for mandibular segmentation:

• Gap closure in dental arches

• Transversal discrepancies

• Asymmetry of the dental arch

• Pro- or retrusion of the lower incisors, often in combination with chin hypoplasia.

Beside BSSO we have performed median osteotomies for both transversal widening and reduction. Segmental osteotomies for the correction of pro- or retrusion of the incisors were indicated very often, sometimes with additional distraction procedures. Often we combined the procedure with a correction of the chin by sliding genioplasty anterior bloc rotation or chin wing.

These corrections facilitated the orthodontic treatment and reduced postsurgical treatment duration. In cases of exact postoperative intercuspation, only dental retention was necessary.

